# Common Bile-Duct Mucosa in Choledochoduodenostomy Patients — Histological and Histochemical Study

**DOI:** 10.1155/1988/85102

**Published:** 1988

**Authors:** E. Eleftheriadis, V. Tzioufa, K. Kotzampassi, H. Aletras

**Affiliations:** 1 Department of Surgery University of Thessaloniki Greece; 2 Department of Pathology University of Thessaloniki Greece; 3 Department of Surgery University of Thessaloniki Thessaloniki GR-54006 Greece

## Abstract

We describe the histological and histochemical changes of the common bile-duct mucosa in specimens obtained by means of peroral cholangioscopy, 1–12 years after choledochoduodenal anastomosis. Our findings — hyperplasia of the superficial epithelium, metaplastic goblet cells containing predominantly acid sialomucins, and pyloric-like gland formation containing neutral mucins — express a morphological and functional differentiation of the common bile-duct mucosa that probably facilitates its survival in a different environment. We consider that these adaptive changes may explain the uneventful long-term postoperative period of choledochoduodenostomized patients.

## INTRODUCTION

After choledochoduodenal anastomosis (CDA), the common bile-duct mucosa (CBDM) is exposed to a different environment, no longer being protected by the sphincter of Oddi. Although, theoretically, this new environment — i.e. gastric acid and food flowing through the anastomosis — should affect it, both clinical practice and experimental data[1] have shown no evidence of disturbance when no stenosis or occlusion is present; in other words, the CBDM "adapts". The capacity of adaption to external circumstances is clearly fundamental to living organisms[2]. During recent years, there has been real progress in the understanding of both the meaning and the mechanisms of adaption, through study of the mucosal morphology and cell kinetics of the small intestine, which represents the most intimate interface between the *milieux exterieur* and *interiur*, and exhibits a remarkable capacity to adapt to environmental changes.

The purpose of this work is the histological and histochemical study of the CBDM — after the CBD has been anastomosed with the duodenum — in an affort to describe the possible adaption changes that occur and the time required for such changes.

## MATERIAL

Our subjects were nine asymptomatic volunteers on whom cholecystectomy and CDA had been performed, 1–12 years (mean ± s.d. = 5.6 ± 4.25) previously, for CBD lithiasis. After being given detailed information, they were subjected to an upper gastro-intestinal endoscopy, with a UGI-$F_3$ Fujinon flexible gastroscope, through which a biopsy from the mucosa of the posterior CBD wall — just opposite the CDA stoma — was performed for histological and histochemical evaluation.

## METHOD

The CBDM specimens were fixed in 10% buffered formalin, embedded in paraffin, cut into 5-μm sections and stained with heamatoxylin–eosin for histological evaluation of the tissue morphology, and with Alcian blue (pH 2.5) – periodic-acid-Schiff[3] and high-iron diamine – Alcian blue (pH 2.5)[4] for histochemical evaluation of the intracellular mucosubstances.

Alcian blue (pH 2.5) – periodic-acid-Schiff distinguishes neutral mucins (staining magenta) from acid mucins (staining blue), and high-iron diamine - Alcian blue (pH 2.5) separates acid mucins into sulphated (staining black-brown) and non-sulfated (staining blue) mucins.

## RESULTS

The CBD is normally lined by simple tall columnar epithelium containing acid-sulfated mucins in the supranuclear area. Small mucous glands are present in the lamina propria, their cell secreting predominantly acid-sulfated mucins, but some secreting neutral and acid sialomucins.

CBDM of all patients exhibited hyperplasia of the superficial epithelial cells forming deep folds and gland-like structures. Occasional metaplastic goblet cells — not normally found in the CBDM — were observed, as well as pyloric-like glands (pseudopyloric glands). Dence inflammatory cell infiltration with lymphocytes, plasma cells and polymorphonuclear cells, and fibrosis were also observed (Figures 1, 2).

Histochemical staining revealed increased secretory activity of the epithelial cells. Metaplastic goblet cells of either the superficial epithelium or the gland-like structures contained predominantly acid sialomucins; some, however, contained neutral or acid-sulfated mucins. Pseudopyloric glands contained neutral mucins, such as the cells of Brunner's gland in the duodenum (Figure 3).

## DISCUSSION

The capacity of an organ or tissue to adapt is expressed by the morphological and functional differentiation that facilitates its survival.

Our findings, i.e. hyperplasia of the superficial epithelium forming villus folds and

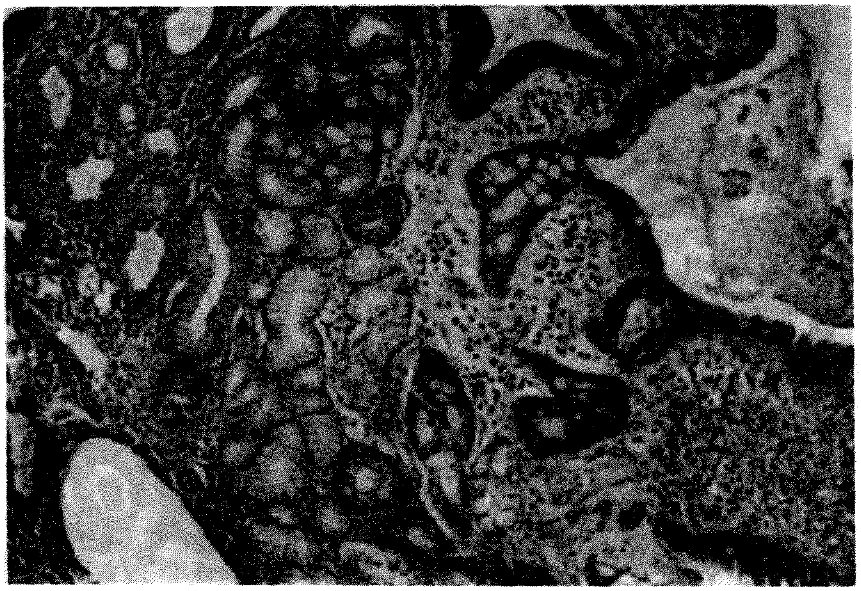

**Figure 1** Common bile-duct mucosa exhibits hyperplasia of the superficial epithelium, pyloric-like gland formation and goblet cells. Dense inflammatory cell infiltration and fibrosis are also present. (HE, X 63)

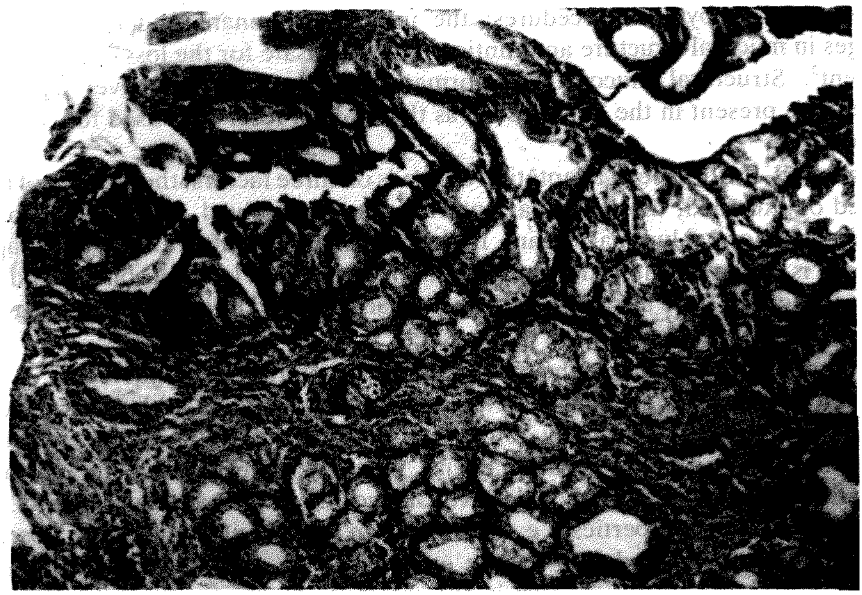

**Figure 2** Common bile-duct mucosa: metaplastic goblet cells and pyloric-like glands are present. (HE, X63)

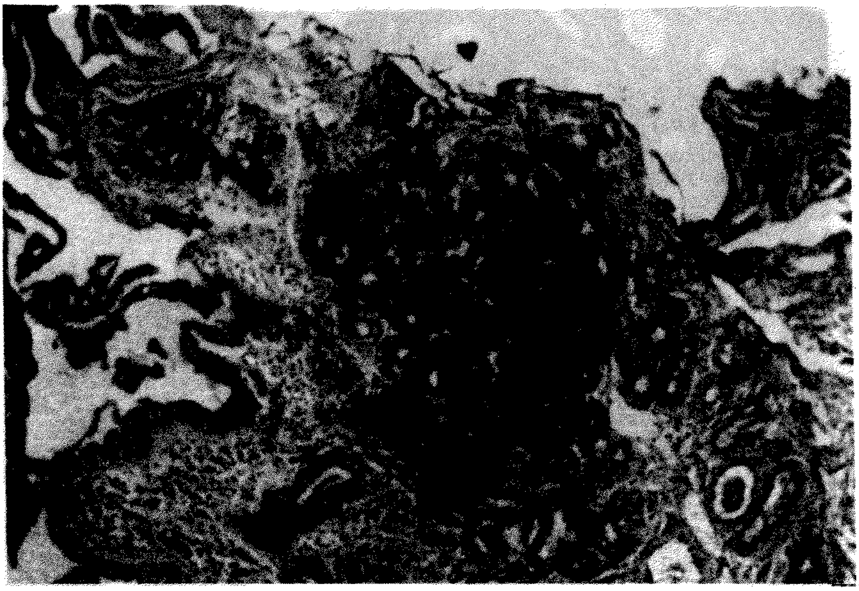

**Figure 3** Common bile-duct mucosa: pyloric-like glands contain neutral mucins, and metaplastic goblet cells contain acid sialomucins. (Alcian Blue pH 2.5–PAS, X 63)

gland-like structures, reveal an increase in the total functional mucosal surface, which is a sign of morphological differentiation. Similar findings are common after enterectomy or bypass procedures; the intestinal remnant undergoes adaptive changes in mucosal structure and funtion to compensate for the loss of the resected segment[5]. Structural mucosal transformation also occurs whenever a noxious influence is present in the lumen, such as that of ileal reservoirs used for continent urostomy[6].

In general, the degree of adaptation depends on three factors: the amount of tissue excised or excluded, the particular enteric segment involved, and the presence or absence of a normal luminal stream. In the case of CBDM after CDA, the adaptive response is due to the alteration in both luminal steam and luminal content and pH[7]. Similar reactions have also been described in airways and intestines after long-term exposure to irritating substances or parasites[8,9].

The most signficant part of the CBD mucosal adaptation is considered by us to be its histological and histochemical metaplasia. The presence of goblet sells secreting acid sialomucins (not normally found in CBDM[10]), in view of the postulated protection afforded by sialic acid against proteolytic degradation in the mucin molecule[11], is evidence that the adaptive changes that occur are "intelligent". The same histochemical properties, i.e. an increase in sialomucin content, are observed in the mucous cells bordering gastric erosions[12]. On the other hand, although Philipson *et al.* report an increase in the number of goblet cells early after ileal urinary reservoir construction — due to the chemical irritation of urine — they consider the mucus released from goblet cells as a non-specific protection[8]. However, it is obvious that the proportions of the different types of mucin produced by the goblet cells vary along the crypts and villi[13]. It is also documented that physiological and environmental factors such as the pH of the medium and bacterial flora may well influence the type of mucin eventually secreted[14].

The pyloric-type gland formation containing only neutral mucins, as Brunner's glands do[15], is evidence of CBD "duodeno-transformation" serving to neutralize the acid chyme from the stomach[16].

Although we attempted to correlate our findings with the time interval since CDA, no such evidence existed. The CBDM structure changes that occur, do so during the first postoperative year. From then on up to the 12th postoperative year, there is no further adaptation. An obvious explanation is that proliferative cell cycle time is extremely rapid[17]. Since there had been a one-year lapse in the case of our most recently operated patient, all adaptive changes in mucosal structure necessary to compensate for the environmental factors had already taken place.

The presence of dense inflammatory cell infiltration and fibrosis observed in the lamina propria is considered to be a physiological reaction due to chronic stimulation from the gastric acid and food refluxing within. Filho *et al.*[18] reported the same findings in the CBD of dogs after experimental sphincterotomy.

We conclude that the adaptive changes observed in CBDM after CDA may explain, at cellular level, the uneventful long-term postoperative period of these patients.

## References

1. Madden, J.L., Chun, J.Y., Kandalaft, S. and Parekh, S. (1970) Choledochoduodenostomy, an unjustly maligned surgical procedure. *Am.J.Surg.*, **119**, 45–54
2. Booth, C.C. (1982) Adaptation. Pathophysiology of intestinal response to disease. Introduction. In *Structure of the Gut,* edited by J.M. Polak, S.R. Bloom, N.A. Wright and M.J. Daly, pp. 317–8. Ware, Hunt, UK: Glaxo Group Research Ltd
3. Pearse, A.G.E. (1968) *Histochemistry, Theoretical and Applied,* Vol. 1, 3rd ed. Edinburgh: Churchill Livingstone
4. Spicer, S.S. (1965) Diamine methods for differentiating mucosubstances histochemically. *J.Histochem.Cytochem.*, **13**, 211–34
5. Dowling, R.H. (1982) Small bowel adaptation and its regulation. In *Structure of the Gut,* edited by J.M. Polak, S.R. Bloom, N.A. Wright and M.J. Daly, pp. 369–90. Ware, Hunt, UK: Glaxo Group Research Ltd
6. Philipson, B.M., Kock, N.G., Jagenburg, R., Ahren, C., Norlen, L., Robinson J.W.L. and Menge, H. (1983) Functional and structural studies of ileal reservoirs used for continent urostomy and ileostomy. *Gut,* **24**, 392–8
7. Bristol, J.B. and Williamson, R.C.N. (1985) Postoperative adaptation of the small intestine. *World J.Surg.*, **9**, 825–32
8. Philipson, B.M., Hockenstrom, T. Akerlund, S. (1987) Biological consequences of exposing ileal mucosa to urine. *World J.Surg.*, **11**,790–97
9. Miller, H.R.P., Nawa, Y. and Parish, C.R. (1979) Intestinal goblet cell differentiation in Nippostrongylus-infected rats after transfer of fractionated thoracic duct lymphocytes. *Int. Arch. Allergy Appl. Immunol.*, **59**, 281
10. Chou, S.T. and Gibson, J.B. (1970) The histochemistry of biliary mucins and the changes caused by infection with *Clonorchis sinensis. J.Pathol.*, **101**, 185–98
11. Gottschalk, A. and de St Groth, S.F. (1960) Studies on mucoproteins. III. The accessibility to trypsin of the susceptible bonds in ovine submaxillary gland mucoprotein. *Biochim. Biophys.Acta,* **43**, 513
12. Lev. R., Molot, M.D., McNamara, J and Stremple, J.F. (1971) "Stress" ulcers following war wounds in Vietnam. A morphologic and histochemical study. *Lab. Invest.*, **25**, 491–502
13. Filipe, M.I. (1979) Mucins in the human gastrointestinal epithelium: a review. *Invest.Cell Pathol.*, **2**, 195–216

14. Florey, H.W. (1970) The secretion of mucus and inflammation of mucus membranes. In *General Pathology,* edited by L. Florey, 4th edn, p.195. London: Lloyd Luke (Medical Books)
15. Florey, H.W. (1962) The secretion and function of intestinal mucus. *Gastroenterology,* **43**, 326–9
16. LaMont, J.T. (1981) Hypersecretion of mucus in gastrointestinal cell injury: possible role in pathophysiology of disease. In: *Basic Mechanisms of Gastrointestinal Mucosal Cell Injury and Protection,* edited by J.W. Harmon pp.391–402. Baltimore: Williams and Wilkins
17. Robbins, S.L. and Cotran, R.S. (1979) *Pathologic Basis of Disease,* p. 954. Philadelphia: W.B. Saunders Co.
18. Filho, E.S. (1986) Histological alterations of the liver, gallbladder and common bile duct after papillotomy. (Abstr. 1506). *Dig.Dis.Sci.,* **31**(Suppl), 379S

*Accepted by S. Bengmark on 17 March 1988*
